# Online In-Tube Solid-Phase Microextraction Coupled to Liquid Chromatography–Tandem Mass Spectrometry for the Determination of Tobacco-Specific Nitrosamines in Hair Samples

**DOI:** 10.3390/molecules26072056

**Published:** 2021-04-03

**Authors:** Atsushi Ishizaki, Hiroyuki Kataoka

**Affiliations:** School of Pharmacy, Shujitsu University, Nishigawara, Okayama 703-8516, Japan; ishizaki@shujitsu.ac.jp

**Keywords:** tobacco-specific nitrosamines, hair, exposure biomarkers, in-tube solid-phase microextraction (SPME), liquid chromatography-tandem mass spectrometry (LC–MS/MS)

## Abstract

Active and passive smoking are serious public health concerns Assessment of tobacco smoke exposure using effective biomarkers is needed. In this study, we developed a simultaneous determination method of five tobacco-specific nitrosamines (TSNAs) in hair by online in-tube solid-phase microextraction (SPME) coupled to liquid chromatography-tandem mass spectrometry (LC–MS/MS). TSNAs were extracted and concentrated on Supel-Q PLOT capillary by in-tube SPME and separated and detected within 5 min by LC–MS/MS using Capcell Pak C18 MGIII column and positive ion mode multiple reaction monitoring systems. These operations were fully automated by an online program. The calibration curves of TSNAs showed good linearity in the range of 0.5–1000 pg mL^–1^ using their stable isotope-labeled internal standards. Moreover, the limits of detection (*S/N* = 3) of TSNAs were in the range of 0.02–1.14 pg mL^–1^, and intra-day and inter-day precisions were below 7.3% and 9.2% (*n* = 5), respectively. The developed method is highly sensitive and specific and can easily measure TSNA levels using 5 mg hair samples. This method was used to assess long-term exposure levels to tobacco smoke in smokers and non-smokers.

## 1. Introduction

Active and passive smoking are serious public health concerns because they increase the risk of various cancers, cardiovascular diseases and respiratory diseases [1,2]. A 2020 study by the World Health Organization (WHO) reported that tobacco kills up to half of the world’s 1.3 billion tobacco users, with active and passive smoking estimated to kill about 7 million and 1.2 million people per year, respectively [3,4]. In particular, persons with passive smoking have been reported to have a 1.3-fold higher risk of developing lung cancer than those without passive smoking [5]. To prevent the health hazards caused by active and passive smoking, it is essential to understand the exposure level to tobacco smoke, and the development of a sensitive and specific method for measuring effective exposure biomarkers is an urgent issue [1,2,6,7,8,9].

Tobacco smoke, which can be broadly classified into gaseous and particulate components, contains about 5300 chemicals, including more than 500 substances associated with mutagenicity and carcinogenesis, such as tobacco-specific nitrosamines (TSNAs) [1,7,8,9,10]. TSNAs are formed by the nitrozation by nitrite and nitric acid of tobacco leaf alkaloids, such as nicotine, nornicotine, anatabine and anabasine, in the process of tobacco production and combustion [10,11,12]. The main TSNAs detected in tobacco products and smoke includes 4-(methylnitrosoamino)-1-(3-pyridyl)-1-butanone (NNK), *N*’-nitrosonornicotine (NNN), *N*’-nitrosoanatabine (NAT), and *N*’-nitrosoanabasin (NAB). NNK, its major metabolite 4-(methylnitrosoamino)-1-(3-pyridyl)-1-butanol (NNAL), and NNN play important roles as cancer inducers [1,8,10]. NNK and NNN are classified as human carcinogens (group 1) in the WHO International Agency for Research on Cancer (IARC), but NAT and NAB as unclassifiable for human carcinogenicity (group 3) [13].

Although the metabolism of TSNAs is not fully understood, about 31% of NNK absorbed into the body is metabolized to NNAL [7,11]. The half-life of NNK is 2.6 h [7], and it is eliminated rapidly from the body after exposure to tobacco smoke. In contrast, the half-life of NNAL is relatively long, ranging from 10 days to 3 weeks in smokers [1,6,9] and 40–45 days in oral tobacco users [1,7,8]. Urinary concentrations of NNAL have been regarded as a biomarker of tobacco smoking and exposure to tobacco smoke [1,6,7,8,9,11]. However, urinary concentrations of these compounds are lower in passive than in active smokers, making them unsuitable for assessing the effects of long-term exposure to environmental tobacco smoke in non-smokers. On the other hand, hair samples have been frequently used to assess and monitor the bioaccumulation due to long-term exposure to exogenous compounds, such as environmental pollutants, drugs and carcinogens [14,15,16,17,18,19], because these compounds can remain trapped in hair shafts and the periods and amount of exposure can be identified depending on their distribution in hair [18]. Another advantage of using hair is that samples can be collected easily and less invasively and can be stored at room temperature for up to five years [7,20]. In addition, the amounts of compounds in the hair are less affected by daily exposure and variations in metabolism than those in other biological matrices [7,21]. However, the levels of TSNAs in the hair are not only significantly lower than those in urine but are much lower in passive smokers than in active smokers [6,7,21,22,23].

All of the sensitive analysis methods reported for TSNAs in the hair are based on liquid chromatography–tandem mass spectrometry (LC–MS/MS) [21,22,23]. These methods are sensitive and specific and useful to identify and quantitate TSNAs in hair, but they require relatively large amounts (20–150 mg) of hair samples. Moreover, sample preparation is both tedious and time-consuming, requiring steps, such as liquid–liquid extraction with dichloromethane and solvent evaporation to dryness, or solid-phase extraction, for separation and preconcentration of TSNAs. We recently developed a simple and sensitive method for the simultaneous determination of four TSNAs, excluding NNAL, in main- and side-stream smoke, involving online in-tube solid-phase microextraction (SPME) coupled to LC–MS/MS [24]. In-tube SPME, using an open tubular capillary column with an inner surface coating as an extraction device, is an efficient sample preparation method that allows automation of the extraction and concentration process and can be easily coupled online to HPLC or LC–MS system using column switching technique [25,26,27]. It not only reduces the use of and exposure to harmful organic solvents but also reduces analysis times and gives higher sensitivity and good precision. We have reported analytical methods for various trace contaminants in hair samples using this technique [28,29,30]. The present study describes the development of an online in-tube SPME LC–MS/MS method for the simultaneous determination of five TSNAs, including NNAL, in hair samples and applying this method to the assessment of tobacco smoke exposure in smokers and non-smokers.

## 2. Results and Discussion

### 2.1. Optimization of In-Tube Solid-Phase Microextraction and Desorption of TSNAs

We previously described the optimization of in-tube SPME conditions for four TSNAs, excluding NNAL [24]. In this study, several parameters, such as type of capillary coating and number and flow-rate of draw/eject cycles, were optimized for 1 ng mL^–1^ each of five TSNAs, including NNAL. Although the peak amount of NNAL was lower than those of the other TSNAs, all five TSNAs could be efficiently extracted into a Supel-Q PLOT capillary by more than 25 repeated draw/eject cycles of 40 μL sample at a flow rate of 0.2 mL min^–1^ (Figures S1 and S2). The absolute extractable amounts of TSNAs onto the capillary column were calculated by comparing peak area counts with the corresponding amount in standard solution directly injected onto the LC columns. Although the extraction yields of NNK, NNN, NAT, NAB and NNAL onto the Supel-Q PLOT column from 1 mL of a standard solution containing 1.0 ng mL^–1^ of each compound were 21.0%, 13.3%, 22.0%, 21.8% and 5.0%, respectively, their coefficients of variation (CVs) were below 5% due to the use of an autosampler. The TSNAs extracted into the stationary phase of the capillary column were dynamically desorbed and introduced directly into the LC column by online mobile phase flow. Since the capillary column was cleaned and conditioned by methanol and mobile phase flow prior to extraction, no carryover of each analyte or matrix component was observed.

### 2.2. LC–MS/MS Analysis of TSNAs and Their Stable Isotope-Labeled Compounds

TSNAs and their stable isotope-labeled compounds were efficiently ionized in the ESI-positive ion mode. The MS/MS operation parameters, including curtain gas, nebulizer gas stream ion spray voltage, the corresponding potentials (DP and EP), CE, and CXP, were optimized for each TSNAs. Under optimum MS/MS conditions, protonated ions [M + H]^+^ (Q1 mass) and prominent fragment ions (Q3 mass) for each compound were detected as precursor and product ions, respectively. The MRM transitions for confirmation and quantification and MS/MS parameters set are shown in Table S1. These data were in good agreement with previously reported data [21,22].

**Figure 1:**
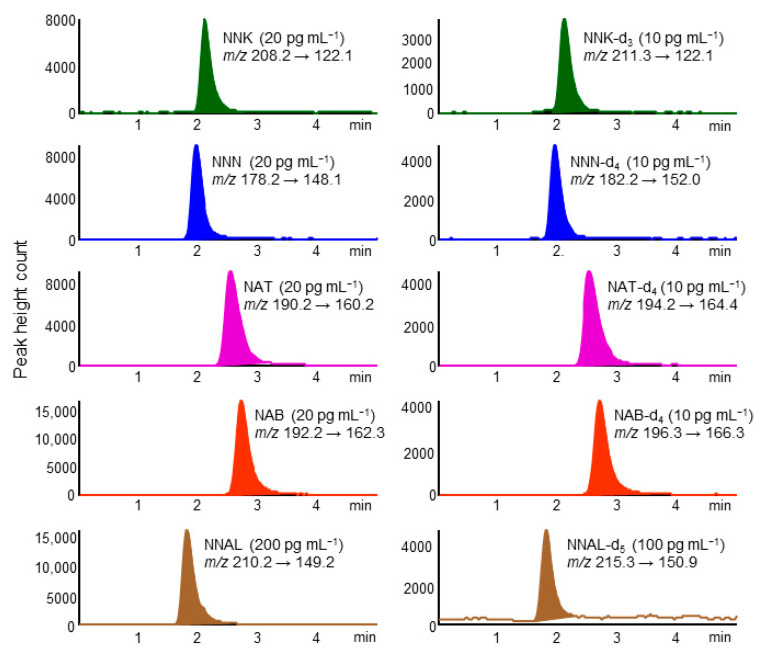
Multiple reaction monitoring (MRM) chromatograms of standard tobacco-specific nitrosamines (TSNAs) and their stable isotope-labeled compounds. In-tube solid-phase microextraction (SPME) LC–MS/MS conditions are described in the Experimental section.

A chromatogram of standard TSNAs by in-tube SPME LC–MS/MS is shown in Figure 1. Five TSNAs and their IS compounds were eluted as well-formed peaks within 4 min on a Capcell Pak C18 MGⅢ column and detected selectively in MRM mode. The CV% of the retention time for each compound was within 5%. The analysis time per sample was about 28 min, allowing automated analysis of about 50 samples per day by operating overnight.

### 2.3. Analytical Method Validation

Linearity was validated by triplicate analyses each for four TSNAs at eight concentrations (0.5, 1.0, 2.0, 5.0, 10, 20, 50, and 100 pg mL^–1^) and for NNAL at eight concentrations (5, 10, 20, 50, 100, 200, 500 and 1000 pg mL^–1^), in the presence of 0.1 ng mL^–1^ each of NNK-d_3_, NNN-d_4_, NAT-d_4_ and NAB-d_4_, and 1 ng mL^–1^ NNAL-d_5_. Calibration curves for each compound were linear with correlation coefficients above 0.9998 (Table 1). The CVs of the peak height ratios at each compound’s concentration ranged from 0.3 to 17% (*n* = 3).

TSNAs gave superior responses in MRM mode detection, with the LODs (*S/N* = 3) of TSNAs in standard solutions ranging from 0.02 to 1.14 pg mL^–1^ (Table 1). The in-tube SPME method was about 11 times more sensitive than the direct injection method (5 µL injection). The LOQs (*S/N* = 10) of TSNAs were 0.02–0.04 pg mg^–1^ hair for all of the TSNAs assayed except NNAL (Table 1). The previously reported LOQs for NNK and NNN were 0.10 and 0.25 pg mg^–1^ hair, respectively [20], indicating that our method’s sensitivity was more than 2.5-fold higher. In contrast, the LOQ of NNAL was 0.75 pg mg^–1^ hair, while that of previous methods ranged from 0.063 to 0.24 pg mg^–1^ hair [20,21].

Precision and accuracy were assessed at low and high concentrations of 2 and 20 pg mL^–1^ for the four TSNAs except for NNAL and 20 and 200 pg mL^–1^ for NNAL. The precision, expressed as CV (%), was validated by performing five independent analyses on the same day and on five different days. The intra-day and inter-day precisions of these analyses were found to be 2.1–7.3% and 3.0–9.2%, respectively (Table 2). Accuracy was validated by comparing the measured concentrations of analytes in samples with the known concentrations of the analyte added to the samples ((found/added) x 100%). The intra-day and inter-day accuracies of these analyses were found to be 94–115% and 96–119%, respectively (Table 2).

These results obtained based on the generally accepted validation criteria recommended in the ICH guidelines [31] show that the method has good linearity, precision and accuracy.

### 2.4. Application to the Analysis of Hair Samples

Since TSNAs absorbed into the body accumulate in the hair, they can be effective biomarkers for evaluating long-term exposure to tobacco smoke. However, internal TSNAs accumulated in the hair must be separated from external TSNAs deposited on the outer surface of the hair before analysis. To remove the external contaminants, the hair samples are usually prewashed with dichloromethane, methanol or 0.1% sodium dodecyl sulfate [14,16,19,22,28]. We ensured that the external TSNAs could be completely removed by washing the hair samples one each with 0.1% sodium dodecyl sulfate, water and methanol. The internal TSNAs in hair samples were easily extracted into distilled water by heating at 80 °C for 30 min, and the extract could be directly used for in-tube SPME/LC–MS/MS without any other pretreatment.

**Figure 2:**
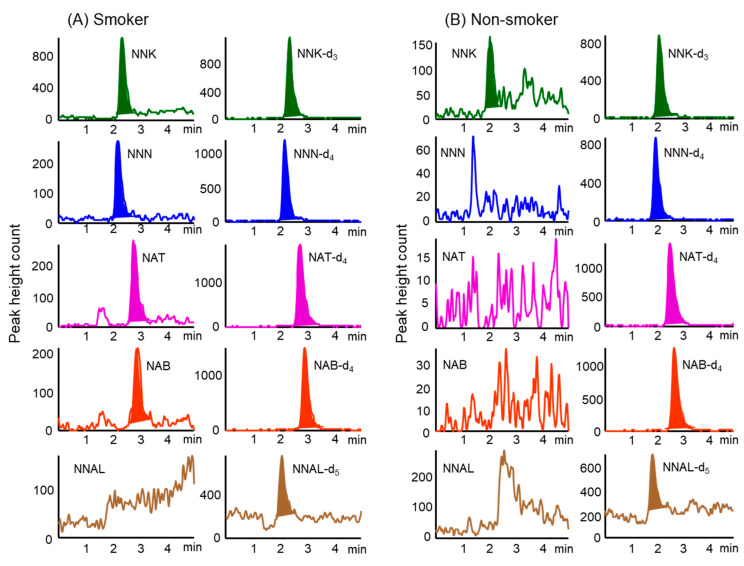
Typical MRM chromatograms were obtained from hair samples of (**A**) smokers and (**B**) non-smoker by in-tube SPME LC–MS/MS. Analytical conditions are described in the Material and Methods section.

Stable isotope-labeled compounds as IS were added to hair samples prior to extraction to minimize the influence of matrix effects on the analysis of TSNAs in the samples. Figure 2 shows typical chromatograms obtained from hair samples (corresponding to 2.5 mg) from smokers and non-smoker. The specificity of this method was verified by analyzing both blank hairs (i.e., TSNA-free) from a non-smoker and the same hair spiked with the five TSNAs to check for co-eluting interferences at the retention times of the compounds of interest. These chromatograms showed no interference with the TSNAs, and their IS compounds from hair samples. In addition, the overall recovery rates of TSNAs spiked into hair samples were over 92% (Table 3).

The developed method was used to analyze TSNA concentrations in hair samples from 25 smokers and 29 non-smokers. Of the five TSNAs assayed, NNK and NNN were present at higher concentrations in hair samples from smokers than the other compounds (Table 4). Although NAT and NBT were often detected at low concentrations, NNAL was not detected at all. In contrast, NNK and NNN were present at low concentrations in hair samples from some non-smokers, whereas NAT, NBT and NNAL were not detected at all.

Although analysis of 150 mg samples of hair from smokers showed the presence of NNAL at concentrations of 0.27–0.67 pg mg^–1^ [22], analysis of 20 mg samples of hair from non-smokers failed to detect any NNAL [21]. Our study, however, found that NNAL was undetectable in hair samples from both smokers and non-smokers. The concentrations of NNK and NNN in the hair samples from smokers were significantly higher than those from non-smokers (*p* < 0.01), and total TSNA concentrations in hair samples were about 20 times higher in smokers than in non-smokers. These results indicate that NNK and NNN in hair are effective biomarkers to assess long-term exposure to tobacco smoke.

## 3. Materials and Methods

### 3.1. Reagents and Standard Solutions

Five standard TSNAs, NNK, NNN, NAT, NAB and NNAL, and their stable isotope-labeled compounds, NNK-d_3_ (isotopic purity 99.9%), NNN-d_4_ (isotopic purity 97.8%), NAT-d_4_ (isotopic purity 98.0%), NAB-d_4_ (isotopic purity 99.7%) and NNAL-d_5_ (isotopic purity 97.8%) as each internal standard (IS) (supplementary Figure S3), were purchased from Toronto Research Chemicals Inc. (North York, ON, Canada). Stock solutions of 1.0 mg mL^–1^ of each compound were prepared by dissolving in LC–MS grade acetonitrile and diluted with distilled water to the required concentration prior to use. The mixed standard solution consisted of 1 ng mL^–1^ each of NNK, NNN, NAT and NAB and 10 ng mL^–1^ NNAL, whereas the mixed IS solution consisted of 0.1 ng mL^–1^ each of NNK-d_3_, NNN-d_4_, NAT-d_4_ and NAB-d_4_, and 1 ng mL^–1^ NNAL-d_5_. These prepared solutions were stored at 4℃. Methanol and distilled water as mobile phases were of LC–MS grade, while all other chemicals were of analytical reagent grade.

### 3.2. Preparation of Hair Samples

Hair samples were provided by 54 healthy Japanese volunteers (46 men and 8 women, aged 23–68 tea), including 25 smokers and 29 non-smokers. Approximately 10 mg of hair was collected from the back of each subject’s head, washed with 0.1% sodium dodecyl sulfate, water and methanol, and stored in an amber glass desiccator at room temperature until use. About 5 mg of hair cut into small pieces with scissors was weighed into a 7 mL screw-cap vial, to which 0.1 mL of water and 0.1 mL of the mixed IS solution were added, and the vial was heated and extracted at 80 °C for 30 min with the cap. The extract was cooled to room temperature and filtered through a 45 μm hydrophilic PTFE syringe filter (Shimadzu GLC Ltd., Tokyo, Japan). For in-tube SPME LC–MS/MS analysis, 0.1 mL of the filtrate was taken into a 2.0 mL autosampler vial with the septum, and the total volume was made up to 0.5 mL with distilled water. The concentrations of each TSNA in hair were calculated using a calibration curve constructed from the ratios of peak heights of each TSNA to the peak heights of their IS compounds.

### 3.3. LC–MS/MS Analysis

LC–MS/MS analysis was essentially performed as described in our previous work [24] using an Agilent Technologies Model 1100 series LC system and an Applied Biosystems API 4000 triple, quadruple mass spectrometer. A Capcell Pak C18 MGⅢ column (100 mm × 2.0 mm, particle size 5 μm; Shiseido, Tokyo, Japan) was used as a separation column. The LC conditions were as follows: column temperature, 40 °C; mobile phase, 5 mM ammonium acetate/methanol containing 0.1% acetic acid (50/50, *v/v*); flow rate, 0.2 mL min^–1^. Electrospray ionization (ESI)–MS/MS conditions were as follows: turbo ion spray voltage and temperature, 5000 V and 600 °C; ion source gases (GS1 and GS2) flows, 50 and 80 L min^–1^; curtain gas (CUR) flow, 40 L mL^–1^, collision gas (CAD) flow, 4.0 L min^–1^. Multiple reaction monitoring (MRM) transitions in positive ion mode and other setting parameters, including dwell time, declustering potential (DP), entrance potential (EP), collision energy (CE), and collision cell exit potential (CXP), are shown in Supplementary Table S1. Analyst Software 1.3.1 (Applied Biosystems, Foster City, CA, USA) was used for LC–MS/MS data analysis.

### 3.4. In-Tube SPME

In tube, SPME was essentially performed as described in our previous works [24,29]. A GC capillary column (60 cm × 0.32 mm i.d.) as an extraction device was connected between the injection needle and injection loop of the autosampler. The capillary column was threaded through a 1/16 inch polyetheretherketone (PEEK) tube with a 2.5 cm long, 330 μm inner diameter and connected using standard 1/16 inch stainless steel nuts, ferrules and connectors. Supel-Q PLOT (Supelco, Bellefonte, PA, USA), Carboxen 1010 PLOT (Supelco), CP-Sil 5CB (Varian Inc., Lake Forest, CA, USA), CP-Sil 19CB (Varian), CP-Wax 52CB (Varian), and Quadrex 007-5 (Quadrex Corporation, Woodbridge, CT) were used to compare extraction efficiencies. The control of extraction, desorption, and injection was programmed by the autosampler software (Table S2) [24,29].

## 4. Conclusions

The automated online in-tube SPME LC–MS/MS method developed in this study enabled continuous extraction and enrichment of five TSNAs and their sensitive and selective simultaneous analysis. The method is easy to apply to the analysis of a few milligrams of hair samples without tedious pretreatment. Therefore, the proposed method can be a useful tool for biomonitoring smoking levels and for assessing long-term exposure to tobacco smoke over days to months.

## Figures and Tables

**Table 1:** Linearity and sensitivity of the in-tube SPME LC–MS/MS method for TSNAs.

TSNA	Linearity		LOD ^2^ (pg mL^–1^)		LOQ ^3^ (pg mg^–1^)
	Range (pg mL^–1^)	Linearity ^1^ (R^2^)	Direct Injection	In-Tube SPME	In-Tube SPME
NNK	0.5–100	0.9999	1	0.05	0.03
NNN	0.5–100	1	1.8	0.04	0.03
NAT	0.5–100	0.9999	1.1	0.02	0.02
NBT	0.5–100	0.9998	3.5	0.07	0.04
NNAL	5–1000	0.9999	12.9	1.14	0.75

^1^ correlation coefficient (*n* = 24). ^2^ Limits of detection: pg mL^–1^ sample solution (signal-to-noise ratio of 3). ^3^ Limits of quantification: pg mg^–1^ hair sample (signal-to-noise ratio of 10).

**Table 2:** Precision and accuracy of the in-tube SPME LC–MS/MS method for TSNAs.

TSNA	Nominal Concentration (pg mL^–1^)	Precision (CV ^1^%) (*n* = 5)		Accuracy (%) (*n* = 5)	
		Intra-Day	Inter-Day	Intra-Day	Inter-Day
NNK	2	3.6	6.4	100.5	102.5
	20	2.7	3.7	100.7	100.3
NNN	2	7.3	9.2	104.5	104.5
	20	2.7	4.6	104.7	104.2
NAT	2	4.4	5.2	113.5	112.0
	20	2.1	3.0	103.9	105.2
NBT	2	3.1	8.0	114.5	118.5
	20	3.0	7.7	94.2	99.3
NNAL	20	4.2	7.6	95.0	96.3
	200	4.2	7.0	94.3	99.2

^1^ CV, coefficient of variation.

**Table 3:** Recoveries of TSNAs spiked into hair samples of a non-smoker.

TSNA	Concentration (pg mg^–1^ Hair)		Recovery
	Spiked	Mean ± SD (*n* = 3)	(%)
NNK	0	ND ^1^	
	2	1.85 ± 0.12	92.3
	20	20.0 ± 0.4	100.2
NNN	0	ND	-
	2	1.95 ± 0.09	97.3
	20	19.0 ± 0.8	94.9
NAT	0	ND	-
	2	1.97 ± 0.07	98.4
	20	20.1 ± 0.2	100.4
NAB	0	ND	-
	2	1.92 ± 0.05	96.2
	20	19.7 ± 0.2	98.3
NNAL	0	ND	-
	20	18.7 ± 0.9	93.7
	200	205.4 ± 0.8	102.7

^1^ not detectable.

**Table 4:** Detection frequencies and contents of TSNAs in hair samples of smokers and non-smokers.

TSNA	Smokers (*n* = 24)					Non-Smokers (*n* = 29)				
	Detection Frequency (%)	Content (pg mg^–1^ Hair)				Detection Frequency (%)	Content (pg mg^–1^ Hair)			
		Mean ± SD	Min.^1^	Med. ^1^	Max. ^1^		Mean ± SD	Min.	Med.	Max.
NNK	100	0.95 ± 0.96 ^2^	0.08	0.68	3.97	34	0.05 ± 0.08 ^2^	0.00	0.00	0.25
NNN	100	0.43 ± 0.85 ^3^	0.02	0.22	4.44	14	0.02 ± 0.06 ^3^	0.00	0.00	0.24
NAT	68	0.09 ± 0.09	0.00	0.08	0.35	0	ND	0.00	0.00	0.00
NBT	88	0.13 ± 0.27	0.00	0.06	1.09	0	ND	0.00	0.00	0.00
NNAL	0	ND ^4^	ND	ND	ND	0	ND	0.00	0.00	0.00
Total	100	1.61 ±1.55 ^5^	0.11	1.08	6.72	34	0.08 ± 0.11 ^5^	0.00	0.00	0.31

^1^ Min., minimum; Med., median; Max., maximum. ^2, 3, 5^ *p* < 0.01, probability (significant difference *t*-test between smokers and non-smokers). ^4^ Not detectable.

## Data Availability

The data presented in this study are available on request from the corresponding author.

## Supplementary Materials

The following are available online, Figure S1: Structures of the five TSNAs and their stable isotope-labeled TSNAs as internal standards; Figure S2: Schematic diagrams of the automated online in-tube SPME/LC–MS/MS system; Figure S3: Effects of capillary coatings on the in-tube SPME of TSNAs. TSNAs were extracted by 30 draw/eject cycles of 40 µL of standard solution (1 ng mL^–1^) at a flow rate of 200 µL min^–1^; Figure S4: Effects of the number of draw/eject cycles on the in-tube SPME of TSNAs. TSNAs were extracted on a Supel-Q PLOT capillary column by the indicated number of draw/eject cycles of 40 µL of standard solution (1 ng mL^–1^) at a flow rate of 200 µL min^–1^; Table S1: MRM transitions and setting parameters for TSNAs and their stable isotope-labeled compounds; Table S2: Program for the in-tube SPME process.

## Sample Availability

Samples of the compounds are not available from the authors.
