# Supplementary material for: Online In-Tube Solid-Phase Microextraction Coupled to Liquid Chromatography–Tandem Mass Spectrometry for the Determination of Tobacco-Specific Nitrosamines in Hair Samples
Source: Molecules. 2021 Apr 3;26(7):2056. doi: 10.3390/molecules26072056 (PMC8038370; doi:10.3390/molecules26072056)
Supplement: Supplementary file 1 [file molecules-26-02056-s001.pdf]

Supplementary Information

Online In-Tube Solid-Phase Microextraction Coupled to Liquid Chromatography–Tandem Mass Spectrometry for the Determination of Tobacco-Specific Nitrosamines in Hair Samples

Atsushi Ishizaki[1], Hiroyuki Kataoka[1,*]

[1]School of Pharmacy, Shujitsu University, Nishigawara, Okayama 703-8516, Japan

Table S1. MRM transitions and setting parameters for TSNAs and their stable isotope-labeled compounds.

TSNA	RT[1] (min)	Mass transition (m/z)	Dwell time (msec)	DP[2] (V)	EP[3] (V)	CE[4] (V)	CXP[5] (V)
NNK	2.1	208.2 →122.1	100	50	10	15	5
NNN	2.0	178.2 →148.1	100	45	10	15	5
NAT	2.6	190.2 →160.2	100	45	10	15	5
NAB	2.7	192.2 →162.3	100	45	10	15	5
NNAL	1.8	210.2 →149.2	100	50	10	15	5
NNK-d_3	2.1	211.3 →122.1	100	50	10	15	5
NNN-d_4	2.0	182.2 →152.1	100	45	10	15	5
NAT-d_4	2.6	194.2 →164.4	100	45	10	15	5
NAB-d_4	2.7	196.3 →163.3	100	45	10	15	5
NNAL-d_5	1.8	215.3 →150.9	100	50	10	15	5

[1]Retention time (min)

[2]Declustering potential (V)

[3]Entrance potential (V)

[4]Collision energy (V)

[5]Collision cell exit potential (V)

Table S2. Program for the in-tube SPME process.

Sequence	Event	Switching valve	Vial	Draw/ejection		
				Cycle[1]	Volume (µL)	Speed (µL min^{-1})
1	Conditioning of the capillary	Load	MeOH	D/E (2)	40	200
2	Drawing of air into the capillary	Load	Empty	D (1)	50	200
3	Conditioning of the capillary	Load	Water	D/E (2)	40	200
4	Extraction of analytes into the capillary	Load	Sample	D/E (30)	40	200
5	Needle washing	Load	MeOH	D/E (1)	2	200
6	Desorption of analytes from the capillary	Inject	–	–	–	–
7	HPLC separation of analytes and return to sequence 1	Load	–	–	–	–

[1] D: draw, E: ejection.

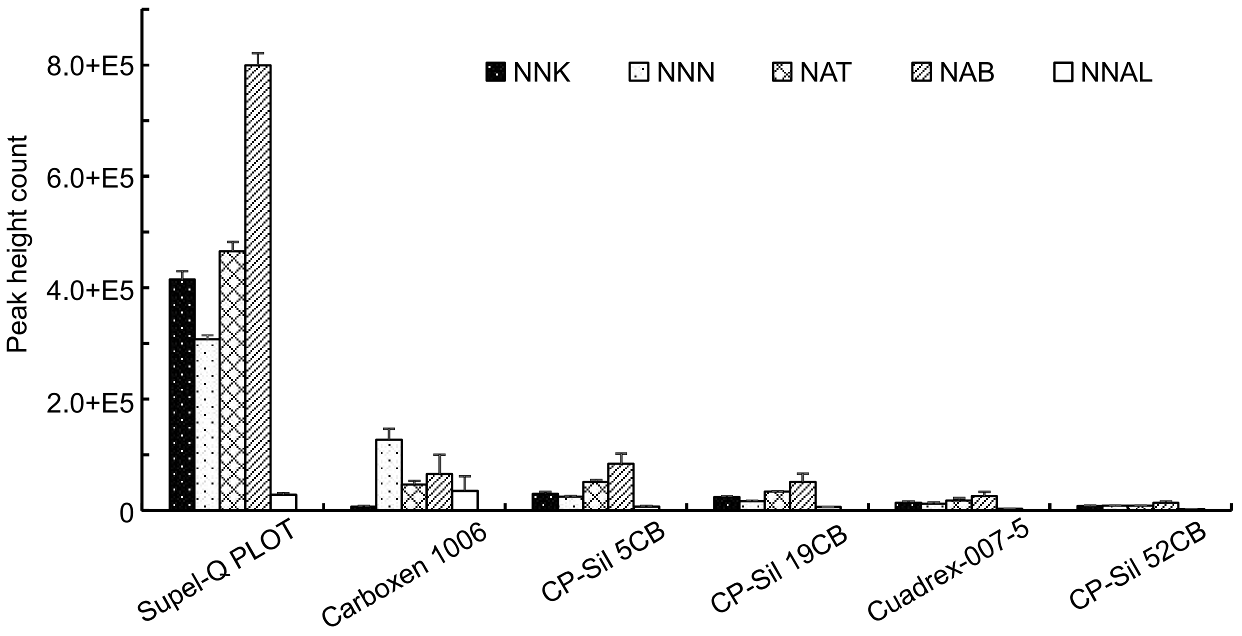

Figure S1. Effects of capillary coatings on the in-tube SPME of TSNAs. TSNAs were extracted by 30 draw/eject cycles of 40 μL of standard solution (1 ng mL^{-1}) at a flow rate of 200 μL min^{-1}.

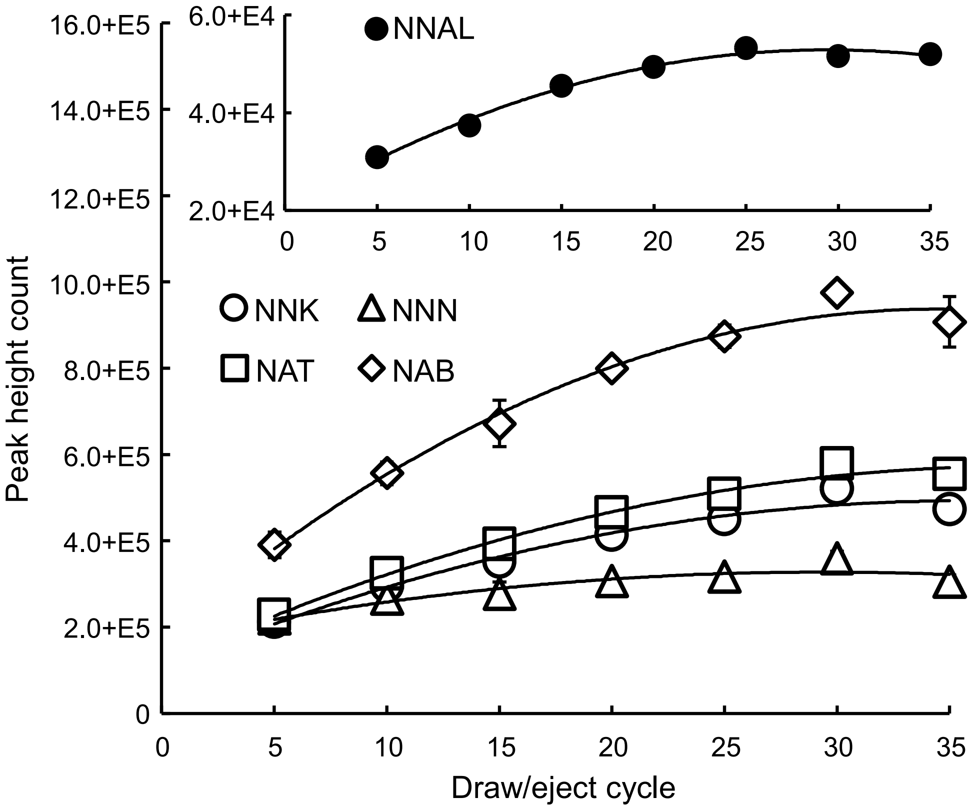

Figure S2. Effects of the number of draw/eject cycles on the in-tube SPME of TSNAs. TSNAs were extracted on a Supel-Q PLOT capillary by the indicated number of draw/eject cycles of 40 μL of standard solution (1 ng mL^{-1}) at a flow rate of 200 μL min^{-1}.

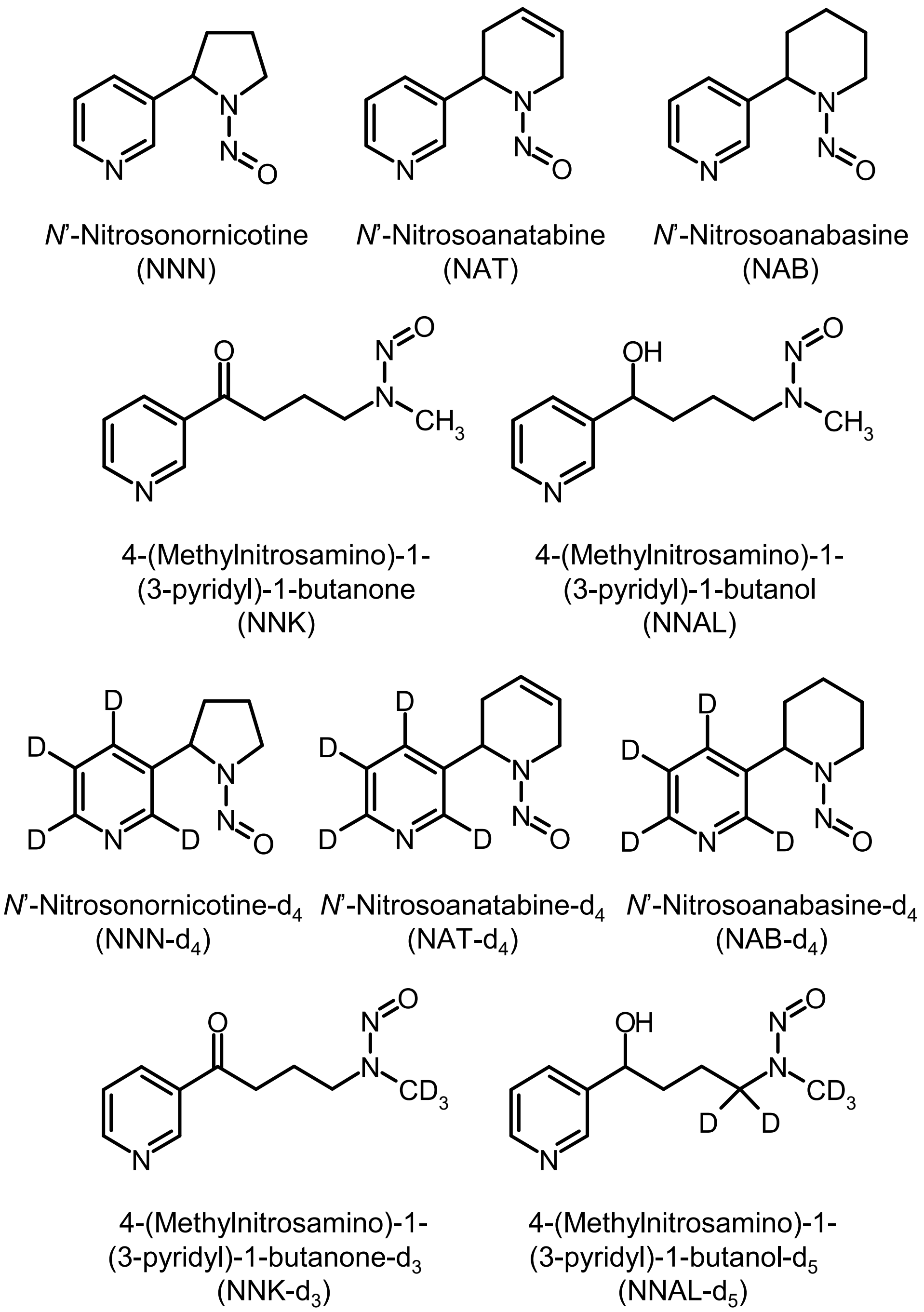

Figure S3. Structures of the five TSNAs assayed and their respective stable isotope-labeled TSNAs as internal standards.
